# A Different Breed of Wolf: First Known Case of Wolf–Hirschorn Syndrome with Third-degree Atrioventricular Block Requiring Pacemaker Implantation

**DOI:** 10.19102/icrm.2020.110601

**Published:** 2020-06-15

**Authors:** Robert Lee Percell, Hyacinth C. Percell, Keith A. Miller

**Affiliations:** ^1^Electrophysiology Section, Bryan Heart Institute, Lincoln, NE, USA

**Keywords:** Arrhythmia, heart block, Wolf–Hirschorn syndrome

## Abstract

We describe a case of a 44-year-old male with a history of Wolf–Hirschorn syndrome (WHS) with seizures and mental retardation who was evaluated for what was thought to be a seizure. He was found to be severely bradycardic with a heart rate of 24 bpm. The electrocardiogram revealed third-degree atrioventricular block and he subsequently underwent an uncomplicated single-chamber pacemaker implantation procedure. This is a unique report given its status as the first described case of bradycardic rhythm abnormalities in a patient with WHS.

## Case presentation

A 44-year-old Caucasian male presented to the clinic as severely disabled and nonverbal at baseline. He had a history of Wolf–Hirschorn syndrome (WSH), seizures, and mental retardation. He was reported to have displayed seizure-like activity that included the shaking of his right side, eyes rolling back into his head, and lips turning blue, which were similar to observed signs of his previous seizures. His last reported seizure was in 2017. His sister stated that she had not noticed any fever or flu-type symptoms. She feeds him baby food and he drinks out of a sippy cup. He is bedridden but can sit in a wheelchair. He had no recent rash or insect or tick exposure. There were no family members with arrhythmias or developmental disorders.

**Figure 1:**
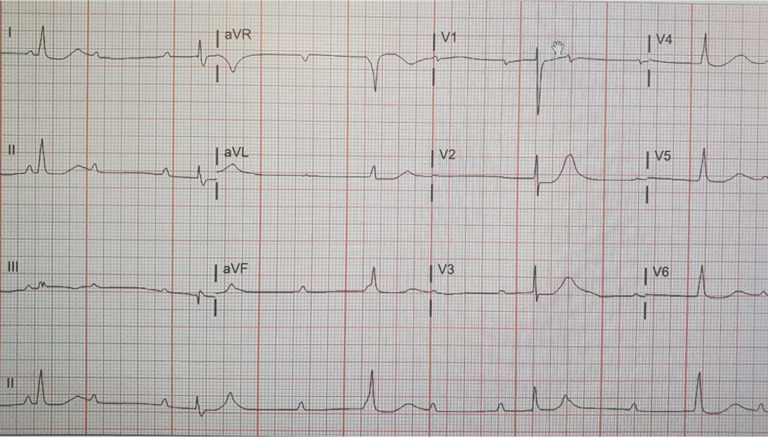
Initial electrocardiogram revealing third-degree AV block with an atrial rate of 75 bpm and a junctional escape rate of 30 bpm with a normal axis.

**Figure 2:**
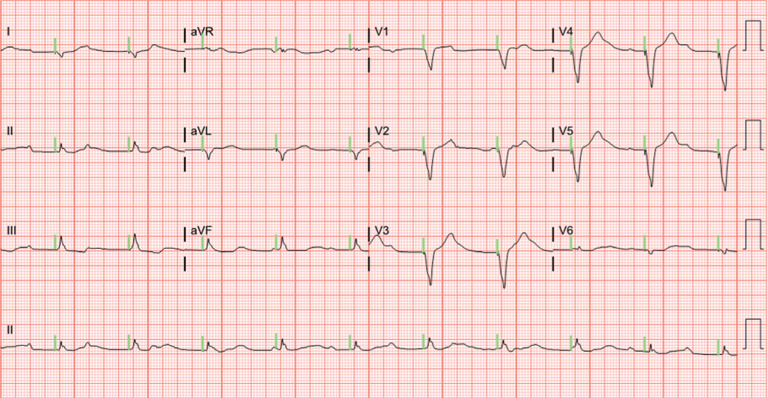
Electrocardiogram showing ventricular pacing with a rate of 60 bpm.

Upon arrival to the emergency department, he was found to be severely bradycardic with a heart rate of 20 to 30 bpm with third-degree atrioventricular (AV) block. The atrial rate was 120 bpm, while the junctional escape rate was 30 bpm **(Figure 1)**. The systolic blood pressure was 90 mmHg. He was given 1 L of intravenous fluids, with some response; his heart rate increased to 60 bpm and his systolic blood pressure increased to 120 mmHg **(Figure 2)**. However, an hour later, his heart rate returned to 20 bpm and his systolic blood pressure was measured as 70 mmHg.

A routine laboratory examination on admission revealed the following: elevated white blood cell count of 24.4 tho/cmm, normal hemoglobin and hematocrit values, sodium level of 135 mEq/L, potassium level of 4.6 mEq/L, blood urea nitrogen level of 34 mg/dL, and creatinine level of 1.8 mg/dL. His troponin and thyroid-stimulating hormone levels were normal, while other laboratory findings were unremarkable. Further, the total valproic acid level was 120 μg/mL (therapeutic range: 50–125 μg/mL). The chest radiograph was unremarkable, with normal cardiac size and clear lung fields.

He was admitted to the intensive care unit and placed on a dopamine drip overnight. The following day, he remained in third-degree AV block. His white blood cell count had decreased to 15.7 tho/cmm, and an echocardiogram revealed normal left ventricular systolic function with an ejection fraction of 60%, with mild to moderate mitral regurgitation. There was no evidence of congenital heart disease.

**Figure 3:**
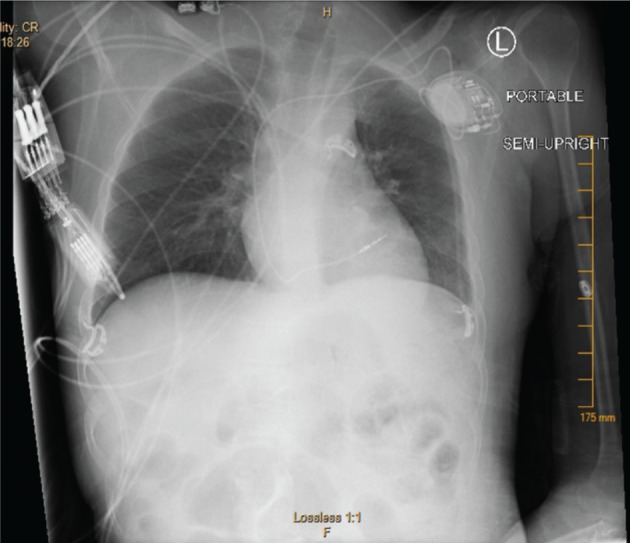
Chest radiograph revealing the patient’s single-chamber pacemaker with the ventricular lead placed in the septum.

After a long discussion with the family, the patient underwent uncomplicated single-chamber pacemaker implantation under general anesthesia **(Figure 3)**. He was discharged two days later.

At the two-week follow-up checkup, the patient was 100% ventricular-paced. His caregiver reported no further episodes of seizure activity.

Unfortunately, two months later, the patient was readmitted with leukocytosis secondary to pacemaker pocket infection. Coagulase-negative staphylococcus grew from both blood and wound cultures. He was treated with cefazolin for a planned six-week course. Transthoracic echocardiogram suggested a bicuspid valve at that point.

**Figure 4:**
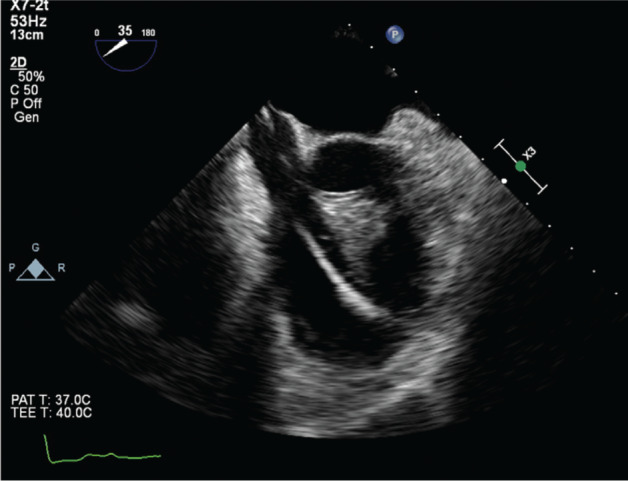
Transesophageal echocardiogram revealing a 3.2-cm × 2.4-cm mass attached to the RV lead, extending to the RV free wall.

One week after discharge, he was readmitted with a seizure and leukocytosis. Transesophageal echocardiogram revealed a definite bicuspid valve as well as extensive vegetation on the single right ventricular (RV) lead that was highly mobile and contacted the RV free wall **(Figure 4)**. After extensive discussions with the guardian with the power of attorney regarding performing further invasive measures including extraction, he was discharged with hospice care.

## Discussion

WHS is a very rare chromosomal disorder that occurs in approximately one in 20,000 to 50,000 newborns.^1^ It is two times more likely to be seen in females and the etiology is the terminal deletion of the short arm of chromosome 4.^2^ The more significant the amount of missing chromosomal material, the larger the clinical expression of WHS that is seen is.^3^ This syndrome was first described by Hirschorn and Cooper.^3^ Wolf et al. later confirmed their findings.^4^

A review of 43 cases of WHS in 1976 reported a mortality rate of 34% in the first two years of life; however, a later study showed that survival increases dramatically after two years of age.^5^ WHS patients frequently die prematurely secondary to lower respiratory-tract infection (25%), multiple congenital anomalies (15.6%), sudden unexplained death (15.6%), congenital heart disease (15.6%), and anoxia at birth (9.4%).^5^ Reported types of congenital heart disease have included one case of tetralogy of Fallot; two with a ventricular septal defect and patent ductus arteriosus; one with an ostium secundum atrial septal defect; and a complex case involving a double-outlet single ventricle with a single AV valve, large aorta, and small posterior pulmonary artery with pulmonary valve stenosis. There is additionally one report of a male in the Netherlands who was 58 years old as of 2014.^6^

The diagnosis of WHS is initially made based on infant facial features and developmental retardation; however, it can be confirmed with genetic testing.^7^ There is no definite gene defect but most experts have identified the following candidate genes: *WHSC1*, *WHC2*, and fibroblast growth factor receptor 3 (*FGFR3*).^8^ The differential diagnosis includes other genetic disorders with microdeletions such as Rubinstein–Taybi syndrome, Smith–Magenis syndrome, and Pitt–Rogers–Danks syndrome.^9^

WHS is frequently described based on the presence of intellectual disabilities; the Greek warrior helmet appearance of the nose and forehead; and multiple other skeletal, cardiovascular, and urogenital defects. Cardiac manifestations are seen in 31% to 45% of cases and are responsible for nearly 35% of deaths in the first year of life.^10,11^ There is no discovered abnormality on chromosome 4 that is associated with AV block. Additionally, no channelopathies have been identified in connection with WHS such as mutations in the *SCN5A* and *TRPM4* genes that have been shown to cause most cases of progressive familial heart block types IA and IB, respectively.^12^

Our patient was diagnosed with WHS during his first year of life, as he displayed decreased muscle tone and classic facial features at that time. Karyotyping revealed a partial deletion of the short arm of chromosome 4. At the time of this case, he had outlived 95% of other patients with this condition. An echocardiogram did not reveal structural or congenital abnormalities. There are no reports in the literature of WHS patients with significant rhythm abnormalities. This may be secondary to the overall decreased longevity. To our knowledge, this is the first reported case of a person with WHS with third-degree AV block who underwent successful pacemaker implantation. A single-chamber VVI device was chosen to minimize the risk of lead dislodgement, as he would not be able to keep the arm immobilized. Consideration of pacemaker syndrome was contemplated, but, as the patient was immobile, this was deemed not to be an issue. In retrospect, this decision was likely the correct one, as the patient developed endocarditis due to single RV lead implantation. Perhaps, the use of a leadless pacemaker may have lessened this risk.
